# Supplementary material for: New and Efficient Bioactive Glass Compositions for Controlling Endodontic Pathogens
Source: Nanomaterials (Basel). 2022 May 6;12(9):1577. doi: 10.3390/nano12091577 (PMC9105659; doi:10.3390/nano12091577)
Supplement: Supplementary file 1 [file nanomaterials-12-01577-s001.zip › nanomaterials-1658589-supplementary.pdf]

SUPPORTING INFORMATION

New and Efficient Bioactive Glass Compositions for Controlling Endodontic Pathogens

Bruna L. Correia [1], Ana T.P.C. Gomes [2], Rita Noites [2], José M.F. Ferreira [1] and Ana S. Duarte [2,*]

[1] Department of Materials and Ceramic Engineering, CICECO-Aveiro Institute of Materials, Campus Santiago, University of Aveiro, 3810-193 Aveiro, Portugal; brunacorreia@ua.pt (B.L.C.); jmf@ua.pt (J.M.F.F.)

[2] Universidade Católica Portuguesa, Faculdade de Medicina Dentária, Centro de Investigação Interdisciplinar em Saúde, 3504-505 Viseu, Portugal; apgomes@ucp.pt (A.T.P.C.G.); rita.noites@ucp.pt (R.N.)

* Correspondence: asduarte@ucp.pt; Tel.: +351-232-419-500

Table S1. Tukey's multiple comparisons test between incubation times with *Candida albicans.*

Samples and Concentrations (mg mL^{-1})		Time	Mean difference	95 % CI of difference		Std. Error of difference	Sig.
				Lower Bound	Upper Bound		
BG1	5.0	**0 h vs 24 h**	0.08	−0.97	1.13	0.32	ns
		**0 h vs 48 h**	−0.24	−1.30	0.81		ns
		**24 h vs 48 h**	−0.32	−1.38	0.73		ns
	15	**0 h vs 24 h**	3.45	2.40	4.51		<0.0001
		**0 h vs 48 h**	3.85	2.80	4.91		<0.0001
		**24 h vs 48 h**	0.40	−0.65	1.45		ns
	Ct	**0 h vs 24 h**	−1.91	−2.96	−0.86		<0.0001
		**0 h vs 48 h**	−4.92	−5.97	−3.87		<0.0001
		**24 h vs 48 h**	−3.01	−4.06	−1.96		<0.0001
BG2	5.0	**0 h vs 24 h**	0.63	0.08	1.18	0.22	p=0.0230
		**0 h vs 48 h**	0.30	−0.25	0.85		ns
		**24 h vs 48 h**	−0.33	−0.88	0.23		ns
	15	**0 h vs 24 h**	3.25	2.70	3.80		<0.0001
		**0 h vs 48 h**	3.97	3.42	4.52		<0.0001
		**24 h vs 48 h**	0.72	0.17	1.27		p=0.0082
	Ct	**0 h vs 24 h**	−2.13	−2.68	−1.58		<0.0001
		**0 h vs 48 h**	−4.92	−5.47	−4.37		<0.0001
		**24 h vs 48 h**	−2.79	−3.34	−2.24		<0.0001
BG3	5.0	**0 h vs 24 h**	0.31	−0.32	0.94	0.26	ns
		**0 h vs 48 h**	0.16	−0.47	0.79		ns
		**24 h vs 48 h**	−0.16	−0.79	0.48		ns
	15	**0 h vs 24 h**	4.04	3.41	4.67		<0.0001
		**0 h vs 48 h**	3.68	3.05	4.31		<0.0001
		**24 h vs 48 h**	−0.36	−0.99	0.27		ns
	Ct	**0 h vs 24 h**	−2.13	−2.76	−1.50		<0.0001
		**0 h vs 48 h**	−4.92	−5.55	−4.29		<0.0001
		**24 h vs 48 h**	−2.79	−3.42	−2.16		ns

Table S2. Tukey's multiple comparisons test between incubation times with *Enterococcus faecalis.*

Samples and Concentrations (mg mL^{-1})		Time	Mean difference	95 % CI of difference		Std. Error of difference	Sig.
				Lower Bound	Upper Bound		
BG1	5.0	**0 h vs 24 h**	−4.58	−6.12	−3.05	0.62	<0.0001
		**0 h vs 48 h**	−3.42	−4.95	−1.89		<0.0001
		**24 h vs 48 h**	1.16	−0.37	2.70		ns
	15	**0 h vs 24 h**	1.41	−0.12	2.95		ns
		**0 h vs 48 h**	−0.39	−1.93	1.14		ns
		**24 h vs 48 h**	−1.81	−3.34	−0.27		*p*=0.0183
	Ct	**0 h vs 24 h**	−4.81	−6.34	−3.27		<0.0001
		**0 h vs 48 h**	−6.16	−7.70	−4.63		<0.0001
		**24 h vs 48 h**	−1.36	−2.89	0.18		ns
BG2	5.0	**0 h vs 24 h**	−5.70	−6.72	−4.68	0.41	<0.0001
		**0 h vs 48 h**	−5.06	−6.08	−4.05		<0.0001
		**24 h vs 48 h**	0.64	−0.38	1.66		ns
	15	**0 h vs 24 h**	1.33	0.31	2.35		*p*=0.0085
		**0 h vs 48 h**	2.45	1.44	3.48		<0.0001
		**24 h vs 48 h**	1.13	0.11	2.15		*p*=0.0271
	Ct	**0 h vs 24 h**	−4.81	−5.83	−3.79		<0.0001
		**0 h vs 48 h**	−6.16	−7.18	−5.15		<0.0001
		**24 h vs 48 h**	−1.36	−2.37	−0.34		*p*=0.0071
BG3	5.0	**0 h vs 24 h**	−4.76	−5.86	−3.65	0.45	<0.0001
		**0 h vs 48 h**	−3.59	−4.70	−2.48		<0.0001
		**24 h vs 48 h**	1.17	0.06	2.28		*p*=0.0371
	15	**0 h vs 24 h**	−0.87	−1.97	0.24		ns
		**0 h vs 48 h**	1.36	0.25	2.47		*p*=0.0134
		**24 h vs 48 h**	2.23	1.12	3.34		<0.0001
	Ct	**0 h vs 24 h**	−4.81	−5.92	−3.70		<0.0001
		**0 h vs 48 h**	−6.16	−7.27	−5.06		<0.0001
		**24 h vs 48 h**	−1.36	−2.46	−0.25		*p*=0.0139

Table S3. Tukey's multiple comparisons test between BGs with *Candida albicans.*

Time	Samples and Concentrations (mg mL^{-1})		Mean difference	95 % CI of difference		Std. Error of difference	Sig.
				Lower Bound	Upper Bound		
0 h	5.0	**Ct vs BG1**	0.27	−0.69	1.23	0.36	ns
		**Ct vs BG2**	0.04	−0.92	1.00		ns
		**Ct vs BG3**	0.20	−0.77	1.16		ns
		**BG1 vs BG2**	-0.23	−1.19	0.73		ns
		**BG1 vs BG3**	-0.08	−1.04	0.89		ns
		**BG2 vs BG3**	0.16	−0.80	1.12		ns
24 h		**Ct vs BG1**	2.26	1.30	3.22	0.36	<0.0001
		**Ct vs BG2**	2.58	1.62	3.54		<0.0001
		**Ct vs BG3**	2.42	1.46	3.38		<0.0001
		**BG1 vs BG2**	0.32	−0.65	1.28		ns
		**BG1 vs BG3**	0.16	−0.80	1.12		ns
		**BG2 vs BG3**	-0.16	−1.12	0.80		ns
48 h		**Ct vs BG1**	4.95	3.99	5.91	0.36	<0.0001
		**Ct vs BG2**	5.26	4.30	6.22		<0.0001
		**Ct vs BG3**	5.27	4.31	6.23		<0.0001
		**BG1 vs BG2**	0.31	−0.65	1.27		ns
		**BG1 vs BG3**	0.33	−0.63	1.29		ns
		**BG2 vs BG3**	0.01	−0.95	0.97		ns
0 h	15	**Ct vs BG1**	0.21	−0.50	0.92	0.27	ns
		**Ct vs BG2**	0.20	−0.51	0.91		ns
		**Ct vs BG3**	0.26	−0.45	0.97		ns
		**BG1 vs BG2**	−0.01	−0.72	0.70		ns
		**BG1 vs BG3**	0.05	−0.66	0.76		ns
		**BG2 vs BG3**	0.06	−0.65	0.77		ns
24 h		**Ct vs BG1**	5.58	4.87	6.28	0.27	<0.0001
		**Ct vs BG2**	5.36	4.65	6.07		<0.0001
		**Ct vs BG3**	6.21	5.50	6.92		<0.0001
		**BG1 vs BG2**	−0.22	−0.93	0.49		ns
		**BG1 vs BG3**	0.63	−0.07	1.34		ns
		**BG2 vs BG3**	0.85	0.15	1.56		p=0.0332
48 h		**Ct vs BG1**	8.99	8.28	9.70	0.27	<0.0001
		**Ct vs BG2**	9.09	8.38	9.80		<0.0001
		**Ct vs BG3**	8.86	8.15	9.57		<0.0001
		**BG1 vs BG2**	0.10	−0.61	0.81		ns
		**BG1 vs BG3**	−0.13	−0.84	0.58		ns
		**BG2 vs BG3**	−0.23	−0.94	0.48		ns

Table S4. Tukey's multiple comparisons test between BGs with *Enterococcus faecalis.*

Time	Samples and Concentrations (mg mL^{-1})		Mean difference	95 % CI of difference		Std. Error of difference	Sig.
				Lower Bound	Upper Bound		
0 h	5.0	Ct vs BG1	0.003	−1.26	1.26	0.47	ns
		Ct vs BG2	0.02	−1.24	1.28		ns
		Ct vs BG3	0.04	−1.22	1.30		ns
		BG1 vs BG2	0.01	−1.25	1.27		ns
		BG1 vs BG3	0.03	−1.23	1.30		ns
		BG2 vs BG3	0.02	−1.24	1.28		ns
24 h		Ct vs BG1	0.23	−1.03	1.49	0.47	ns
		Ct vs BG2	−0.88	−2.14	0.38		ns
		Ct vs BG3	0.09	−1.17	1.35		ns
		BG1 vs BG2	−1.11	−2.37	0.15		ns
		BG1 vs BG3	−0.14	−1.40	1.12		ns
		BG2 vs BG3	0.97	−0.29	2.23		ns
48 h		Ct vs BG1	2.75	1.49	4.01	0.47	<0.0001
		Ct vs BG2	1.12	−0.14	2.38		ns
		Ct vs BG3	2.61	1.35	3.87		<0.0001
		BG1 vs BG2	−1.63	−2.89	−0.37		*p*=0.0021
		BG1 vs BG3	−0.13	−1.39	1.13		ns
		BG2 vs BG3	1.50	0.24	2.76		*p*=0.0332
0 h	15	Ct vs BG1	0.002	−1.27	1.27	0.48	ns
		Ct vs BG2	0.02	−1.25	1.29		ns
		Ct vs BG3	0.03	−1.24	1.30		ns
		BG1 vs BG2	0.02	−1.25	1.29		ns
		BG1 vs BG3	0.03	−1.24	1.30		ns
		BG2 vs BG3	0.01	−1.26	1.28		ns
24 h		Ct vs BG1	6.22	4.96	7.49	0.48	<0.0001
		Ct vs BG2	6.16	4.89	7.42		<0.0001
		Ct vs BG3	3.98	2.71	5.24		<0.0001
		BG1 vs BG2	−0.07	−1.34	1.20		ns
		BG1 vs BG3	−2.25	−3.52	−0.98		*p*=0.0002
		BG2 vs BG3	−2.18	−3.45	−0.91		*p*=0.0002
48 h		Ct vs BG1	5.78	4.51	7.04	0.48	<0.0001
		Ct vs BG2	8.64	7.37	9.91		<0.0001
		Ct vs BG3	7.56	6.29	8.83		<0.0001
		BG1 vs BG2	2.87	1.60	4.14		<0.0001
		BG1 vs BG3	1.78	0.52	3.05		*p*=0.0021
		BG2 vs BG3	−1.08	−2.35	0.18		ns
